# Organic silicon protects human neuroblastoma SH-SY5Y cells against hydrogen peroxide effects

**DOI:** 10.1186/1472-6882-14-384

**Published:** 2014-10-08

**Authors:** Alba Garcimartín, José J Merino, Maria Pilar González, Maria Isabel Sánchez-Reus, Francisco J Sánchez-Muniz, Sara Bastida, Juana Benedí

**Affiliations:** Depatamento de Farmacología, Facultad de Farmacia, Universidad Complutense de Madrid, 28040 Madrid, Spain; Departamento de Nutrición y Bromatología I (Nutrición), Facultad de Farmacia, Universidad Complutense de Madrid, 28040 Madrid, Spain; Departamento de Bioquímica y Biología Molecular II, Facultad de Farmacia, Universidad Complutense de Madrid, 28040 Madrid, Spain

**Keywords:** Silicon, Cellular death, LDH, ROS, Caspase-3,-8,-9, Neuroprotection

## Abstract

**Background:**

Hydrogen peroxide (H_2_O_2_) is a toxic agent that induces oxidative stress and cell death. Silicon (Si) is a biological element involved in limiting aluminium (Al) absorption with possible preventive effects in Alzheimer’s disease. However, Si has not yet been associated with other neuroprotective mechanisms.

**Methods:**

The present experiments evaluated in the SH-SY5Y human neuroblastoma cell line the possible role of different Si G5 (50-1000 ng/mL) concentrations in preventing cellular death induced by H_2_O_2_ (400 μM, 24 hours).

**Results:**

Our findings showed that H_2_O_2_ promoted cell death in the human SH-SY5Y cell cultures and this could be prevented by Si treatment. The loss in cell viability mediated by H_2_O_2_ was due to an apoptotic and necrotic process. Apoptotic death was incurred by regulating caspase-8 activity in the extrinsic pathway. The apoptotic and necrotic cell death induced by H_2_O_2_ was almost totally reversed by Si (50-500 ng/mL), indicating that it down-regulates both processes in H_2_O_2_ treated cells.

**Conclusions:**

According to our data, Si is able to increase SH-SY5Y cell survival throughout partially blocking cellular damage related to oxidative stress through a mechanism that would affect H_2_O_2_/ROS elimination.

## Background

Silicon (Si) is a biologically important element that is soluble in water as silicic acid, Si(OH)_4_. It has been reported that Si is a decisive factor in limiting absorption of dietary Aluminium (Al) [1]. Si administration reduced Al accumulation in several tissues, including brain areas of rats orally exposed to Al [2, 3]. In the form of silicon dioxide (SiO_2_), Si is the most abundant element on the Earth’s crust together with oxygen. Nevertheless, its biological role has not yet been well studied [4]. Si has been considered an “essential” element since chicks receiving Si-deprived diet showed poor growth [5], and because it is also necessary to assure normal growth impairment [6]. Apart from its physiological role in bone and cartilage formation, Si has been associated with cardiovascular protection, immune system enhancement and protective effects in the Alzheimer’s disease [7, 8]. Despite its numerous properties, its biological function remains unclear. Its blood concentration is similar to other elements like zinc [9] and in urine the concentration is like that of calcium, but contrary to other elements and although it is quite ubiquitous [10], it does not associate with plasma proteins [11] and does not have an identified binding site [12]. Finally, Si does not seem to take part in any biochemical reactions/interactions because it does not have bioorganic-inorganic chemistry [13]. Ortho-silicic acid, or monomeric Si(OH)_4_, is water soluble and stable in highly diluted aqueous solutions. It plays a crucial role in delivering Si to the living organisms’ cells. Thus, it represents the major source of Si for both humans and animals. The recommended daily Si intake (RDI) has not yet been set [14, 15]. Some studies suggest that serum Si levels decrease in pregnancy [16] and with aging, particularly in women [17]; thus, supplementation with Si in available forms, such as organic Si, could prevent degenerative processes. As already commented, Si reduces Al bioavailability, thereby possibly limiting Al neurotoxicity. Consumption of moderately large amounts of beer and ortho-silicic acid, in both humans and mice, reduced Al uptake from the digestive tract and prevented the accumulation of Al in the brain [1, 18]. Silicic acid has also been found to induce down-regulation of endogenous antioxidant enzymes associated with Al administration and to normalize tumour necrosis factor alpha (TNF-α) mRNA expression [19]. Davenward et al. [20] showed that Si-rich mineral waters can be used as a non-invasive method to reduce the body burden of Al in both Alzheimer’s patients and a control group by facilitating the removal of Al via the urine without any other concomitant effect. Si has also been shown to induce clinically relevant cognitive improvements in a pilot study with 3 of 15 patients with Alzheimer’s disease [20–22]. This suggests a possible use for ortho-silicic acid as long-term non-invasive therapy for Al reduction in Alzheimer’s disease patients.

To the best of our knowledge, the effect of organic Si on oxidative stress induced by H_2_O_2_ pre-treatment of SH-SY5Y cells has not been evaluated. The present paper hypothesized that Si plays a neuroprotective role against the H_2_O_2_ oxidative aggression by removing ROS. It has been analysed whether organic Si G5 could induces protective effects against H_2_O_2_ neurotoxicity in human neuroblastoma SH-SY5Y cell line. Cellular viability on the MTT assay, caspase activation (caspase-3,-8,-9) and LDH release were all quantified to evaluate the effects of Si and H_2_O_2._ In the present study H_2_O_2_ was chosen as toxic agent because: a) it is the most stable of the long-lived reactive oxygen species (ROS) [23]; b) it passes through cellular membranes [24]; and c) free radicals are involved in the pathogenesis of many degenerative diseases such as Alzheimer’s, Parkinson’s and Multiple sclerosis [25].

## Methods

### Reagents/materials

Silicium organique G5 “TM” was purchased from Glycan Group (Geneva Switzerland). Dulbecco’s modified Eagle’s medium (DMEM), foetal bovine serum (FBS), 0.25% trypsin-EDTA, and penicillin/streptomycin mixture were obtained from GIBCO–BRL (Grand Island, NY, USA). Hydrogen peroxide (H_2_O_2_), 3-(4,5-dimethyl thiazol-2-yl)-2,5-diphenyltetrazoliumbromide (MTT), Ac-DEVD-AMC-[N-acetyl-Asp-Glu-Val-Asp-(7-amino-4-methyl-coumarin)], Ac-LETD-AFC-[Leu-Glu-Thr-Asp-(7-amino-4-trifluoromethyl-coumarin)], Ac-LEHD-AFC-[Leu-Glu-His-Asp-(7-amino-4-trifluoromethyl-coumarin)], barbituric acid, butylated hydroxytoluene (BHT),2,7-dichlorodihydrofluorescein diacetate (H_2_DCF-DA), dimethyl sulfoxide (DMSO), DL-Dithiothreitol (DTT) and 1,1,3,3-tetramethoxypropane were purchased from Sigma Chemical Co. (St. Louis, MO, USA). Other chemicals were reactive grade products from Merck (Darmstadt, Germany).

### Cell culture and treatment

Human SH-SY5Y neuroblastoma cells were grown in DMEM supplemented with 10% foetal bovine serum (FBS) and 100 IU/ml penicillin/streptomycin, maintained in a humidified atmosphere of 5% CO_2_ and 95% air at 37°C. The culture medium was removed every three days and sub-cultures were used once they reached 80–90% confluence. 24 h later, cells were treated in DMEM containing 1% FBS in the presence/absence of organic silicon (50 ng/mL – 20,000 ng/mL), H_2_O_2_ 400 μM or both, together and added at the same time. H_2_O_2_ was freshly prepared from a 30% stock solution prior to each experiment. Control cells without Si treatment were included in all experiments. Treatment was maintained for 24 h except in the experiment of ROS measurement in which cells were treated for only 2 h.

### Protein estimation

The protein concentrations of cell extracts were determined by the Bradford [26] method using bovine serum albumin as standard.

### MTT assay

This assay is based on the ability of living metabolically active cells to reduce the MTT, a soluble formazan salt, given a purple colour. Therefore, the conversion only occurs in living cells. The MTT assay provides a sensitive measurement of the metabolic status of cells and is used to evaluate cell viability. SH-SY5Y were seeded into 96-well culture plates (3×10^4^ cells/well) and allowed to attach. After the above treatment MTT, solution (2 mg/mL) was added to each well and incubated in the dark for 1 hour at 37°C. Supernatants were removed, blue formazan crystals were dissolved in DMSO and absorbance at 595 nm was measured with a microplate reader (LT-4000, Labtech International Ltd, United Kingdom). All MTT assays were performed in quadruplicate. The results are expressed as the percentage of MTT reduction relative to control cells.

### LDH release measurement

Intracellular enzyme lactate dehydrogenase is released into the cell culture medium when cell membrane damage occurs. LDH release is a good marker to detect necrosis. Enzyme activity was determined by spectrophotometric assay according to López et al. [27]. Briefly, NADH oxidation produces a decrease in the absorbance at 340 nm, and this was measured to determined LDH activity by the following formula:


SH-SY5Y cells (3×10^5^ cells/well in 24-well plates) were treated and incubated 24 h at 37°C. Culture medium from all samples was collected. In addition, the cells were homogenized with 0.1 M KH_2_PO_4_/K_2_HPO_4_ (pH 7.4), containing 0.1% Triton X-100. Cells homogenates were centrifuged at 13,000 g for 10 min. LDH activity was measured both in cell supernatant as well as in the culture medium. Activity of the LDH release is expressed as percentage with respect to the total LDH content (LDH in the supernatant + LDH inside the cells) according to the following formula:


### Measurement of reactive oxygen species (ROS) formation

To assay ROS formation, 2,7-dichlorodihydrofluorescein diacetate (H_2_DCF-DA), a non-fluorescent lipophilic reagent, was used. H_2_DCF-DA enters cells, where it is transformed into 2,7-dichlorodihydrofluorescein (H_2_DCF) by the action of intracellular stearases. H_2_DCF is oxidized to fluorescent DCF by H_2_O_2_. H_2_DCF-DA (5 μM) was added to the cells, before subjecting the cultures to different conditions. After 30 min the medium was removed and the cells were treated with Si or/and H_2_O_2_ dissolved in glucose PBS for only 15 min. The fluorescence was measured every 15 min during 2 h in an FL600-BioTek spectrofluorometer (Bio-Tek Instruments INC, Germany) with filters of 485/20 nm excitation and 528/20 nm emission. Results are expressed as arbitrary fluorescence units (AFU).

### Lipid peroxidation assay

Lipid peroxidation was estimated by measuring thiobarbituric acid reactive substances (TBARS) after addition of BHT as antioxidant [28]. Cells were normally treated and the extract was prepared with a lysis buffer (0.1 M KH_2_PO_4_/K_2_HPO_4_ (pH 7.4), containing 0.1% Triton X-100). Briefly, the mixture including 500 μL cell extract, BHT (0.01%), phosphoric acid (1% v/v) and barbituric acid (0.6%) was incubated at 100°C for 45 min. After cooling, 200 μL of the mixture was read at 485/20 nm excitation and 528/20 nm emission wavelengths in a fluorescence plate-reader (FL800, Bio-Tek Instruments INC, Germany). Quantification was performed with a standard curve of malondialdehyde (MDA) generated by an acid hydrolysis of 1,1,3,3- tetramethoxypropane.

### Caspase -3,-8 and -9 activity measurements

SH-SY5Y cells were seeded into 24-well culture plates (3×10^5^ cells/well). 24 h after treatment cells were lysed with lysis buffer (10 mM Tris–HCl, 10 mM NaH_2_PO_4_/Na_2_HPO_4_, pH 7.5, 130 mM NaCl, 0.5% Triton X-100, 10 mM Na_4_P_2_O_7_ and 2 mM DTT) and centrifuged at 13,000 g for 5 min. Caspase-3 activity was measured in the supernatants. Supernatants with at least 20 μg of protein were incubated at 37°C for 1 to 6 hours in caspase-3 assay buffer (20 mM, HEPES, pH 7.5, with 2 mM DTT) and 20 mM Ac-DEVD-AMC [N-acetyl-Asp-Glu-Val-Asp-(7-amino-4-methyl-coumarin)]. Fuorochrome 7-amino-4-methyl-coumarin (AMC) is released from the substrate Ac-DEVD-AMC after reacting with caspase-3 enzyme. The fluorescence signal produced by free AMC is proportional to the caspase-3 activity present in the sample, for at least the first 10 h, and this was monitored using a fluorescence plate-reader (FL800, Bio-Tek Instruments INC, Germany) at 360/40 nm excitation and 460/20 nm emission wavelengths. Enzymatic activity is expressed as arbitrary fluorescence unit after 1 h per mg protein (AFU/h/mg protein).

Caspase-8 and -9 activities were measured as caspase-3 but using their selective substrates (Ac-LETD-AFC-[Leu-Glu-Thr-Asp-(7-amino-4-trifluoromethyl-coumarin)] for caspase-8 and Ac-LEHD-AFC-[Leu-Glu-His-Asp-(7-amino-4-trifluoromethyl-coumarin)] for caspase-9. The fluorochrome released is 7-amino-4-trifluoromethyl-coumarin (AFC), and was measured at 360/40 nm excitation and 530/25 nm emission wavelengths.

### Evaluation of the TNF-α

Release of TNF-α was measured in culture medium after 24 h of treatment using enzyme-linked immunoabsorbent assay (ELISA) (Human TNF-α ELISA Kit, 950.090.096, Diaclone, France) according to the manufacturer’s manual. The increase in colour intensity was evaluated at 450 nm using a microplate reader (LT-4000, Labtech International Ltd, Acorn House, East Sussex, United Kingdom).

### Statistical analysis

Data are shown as mean ± SEM from either two or four independent experiments using different cultures, each experiment performed in triplicate with different cell batches (total 6–12 measurement/condition). Statistical analyses were made with a One-way ANOVA followed by multiple comparisons when the P value was significant. The Dunn’s test was chosen because there were at least six different values per group. A value of p < 0.05 was considered statistically significant. Statistical analyses were performed using Sigma Plot 11.0 software.

## Results

### Action of Si in cell viability

**Figure 1:**
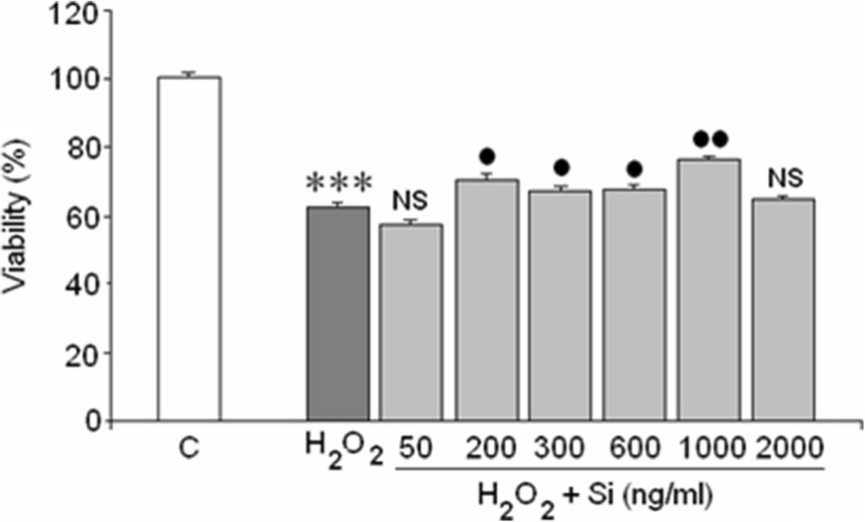
**Effect of H**_**2**_ **0**_**2**_ **in absence/presence of silicon (Si) G5.** Results are expressed as means ± SEM of two experiments from different cultures, each one performed in quadruplicate with different batches of cells (total 8 measurements/condition). (*) = Statistical significances are referred to the basal values. (***) = p < 0.001. (●)/NS Statistical significances between H_2_O_2_ in absence and presence of silicon G5. NS = non significant, (●) = p < 0.05 and (●●) = p < 0.01.

Cells exposed to 400 μM H_2_O_2_ during 24 h showed a significant of decreased (40%) cellular viability. This decline was, in part, reversed by Si G5 at concentrations ranging from 200 to 1000 ng/mL (Figure 1). The lack of a 40% cellular viability induced by H_2_O_2_ in the MTT assay could reflect two events: 1) cellular death or 2) loss of metabolic ability. In order to check these options, LDH release, which measures necrotic death and caspase-3 increase (sensors of apoptosis), were quantified under our experimental conditions.

### LDH assay

**Figure 2:**
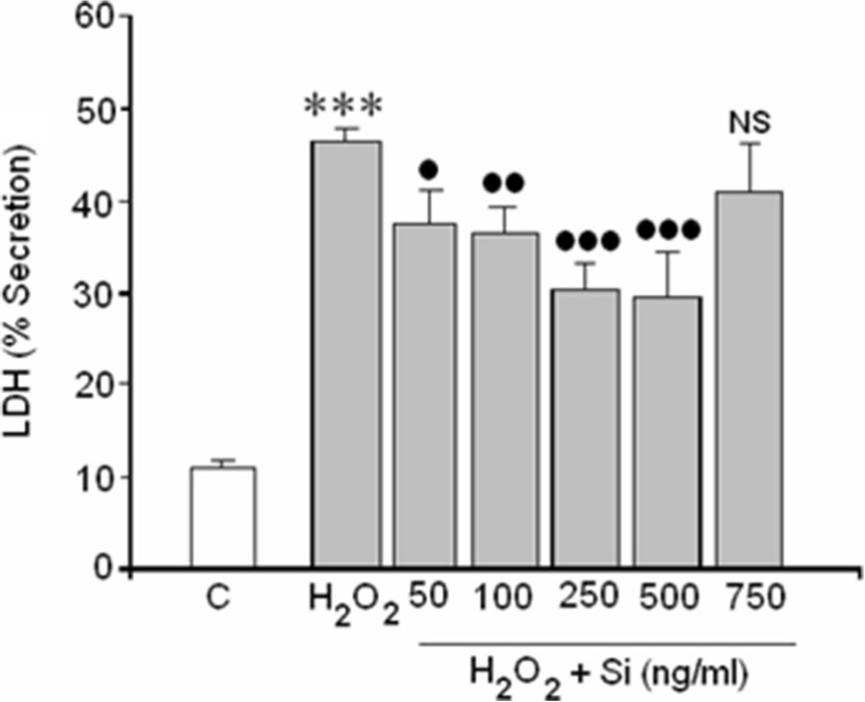
**Effect of Si on LDH release mediated by H**_**2**_ **O**_**2**_ **.** (*) Statistical significances are referred to the basal values. (***) = p < 0.001, (●)/NS = Statistical significances between H_2_O_2_ in absence and presence of silicon G5. NS = non significant, (●) = p < 0.05 and (●●) = p < 0.01. (●●●) = p < 0.001.

Results from Figure 2 show that adding 400 μM of H_2_O_2_, to the cells culture for 24 h, induced significant (p < 0.001) increase of LDH release. This release was partially reversed by Si G5 in a dose dependent manner at concentrations ranging from 50 to 500 ng/mL but never returned to basal values. The highest Si concentration tested (750 ng/mL) had no effect on necrotic death mediated by H_2_O_2_ in this human neuroblastoma cell line.

Since necrotic death can be a consequence of lipid peroxidation and ROS production, both markers were also evaluated.

### Action of Si on ROS formation

**Figure 3:**
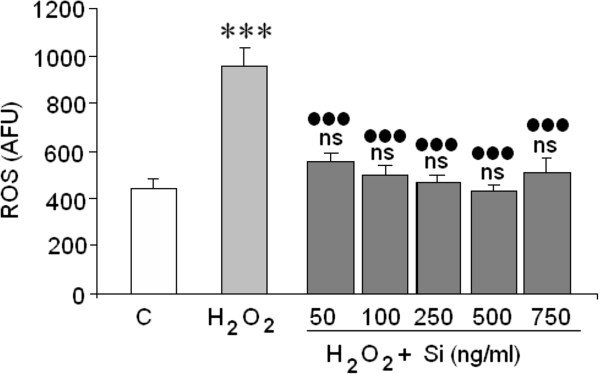
**Effect of Si on H**_**2**_ **O**_**2**_ **(ROS).** (*)/ns = Statistical significances are referred to the basal values. ns = no significant, (***) = p < 0.001, (●) = Statistical significances between H_2_O_2_ in absence and presence of silicon G5. (●●●) = p < 0.001.

Results from Figure 3 demonstrate that Si totally removed ROS from neuroblastoma cells treated with 400 μM H_2_O_2_.

### Effect of Si on lipid peroxidation mediated by H_2_O_2_

**Figure 4:**
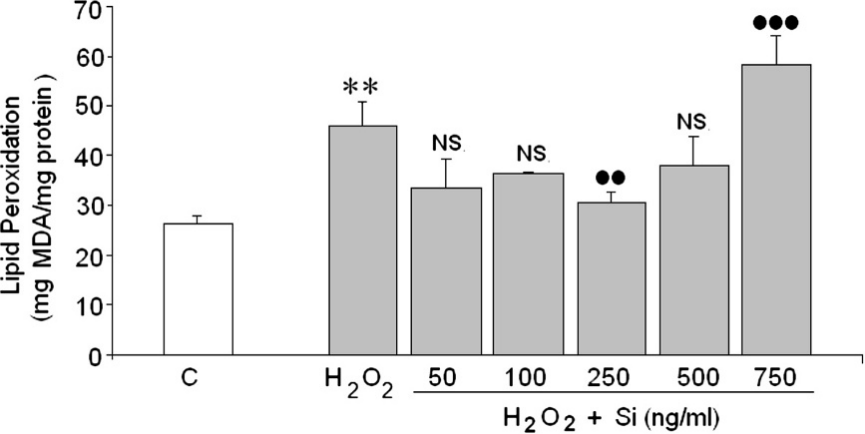
**Effect of Si on lipid peroxidation.** (*) = Statistical significances are referred to the basal values. ns = no significant, (**) = p < 0.01, (●)/NS = Statistical significances between H_2_O_2_ in absence and presence of silicon G5. NS = non significant, (●●) = p < 0.01. (●●●) = p < 0.001.

400 μM H_2_O_2_ increased two-fold lipid peroxidation in the human neuroblastoma cell line (SH-SY5Y). Si at 250 ng/mL reduced the H_2_O_2_ mediated lipid peroxidation to a great extent (Figure 4). However, higher concentrations of Si (750 ng/mL) strongly induced lipid peroxidation which reached values higher than with H_2_O_2_.

### Si action on death mediated by caspase-3

**Figure 5:**
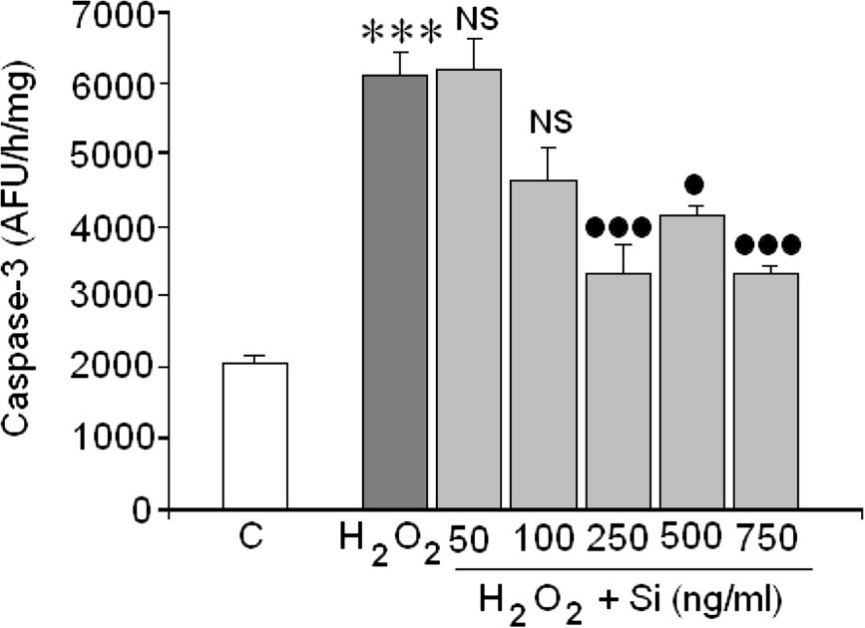
**Action of Si on caspase-3 activation mediated by H**_**2**_ **O**_**2**_ **.** (*) = Statistical significances referred to the basal values. (***) = p < 0.001. (●)/NS = Statistical significances between H_2_O_2_ in absence and presence of silicon G5. (●) = p < 0.05 and (●●●) = p < 0.001.

Once it was found that H_2_O_2_ could induce cellular death through necrosis, the possibility that H_2_O_2_ could also induce cell death through apoptosis was analysed. Figure 5 shows H_2_O_2_ was a potent apoptotic inductor in the SH-SY5Y cell line. Interestingly, Si G5 at concentrations ranging from 250 to 750 ng/mL almost totally reversed the caspase-3 activity that had been increased by H_2_O_2_.

Since caspase-3 can be activated through intrinsic as well as extrinsic pathways, caspase-9 activity (activated by mitochondrial dysfunction), as well as caspase-8 (activated through death receptors) were tested in the present study.

### Action of H_2_O_2_ on caspase-9 activity

Caspase-9 is activated by mitochondrial dysfunction through the release of cytochrome-c which binds to cytosolic protein (Apaf-1) and procaspase-9. In the presence of ATP, a complex that converts inactive procaspase-9 to active caspase-9 is formed. No caspase-9 activation could be observed in the H_2_O_2_-exposed SH-SY5Y cell culture (data not shown).

### Effect of Si on H_2_O_2_-mediated caspase-8 activity

**Figure 6:**
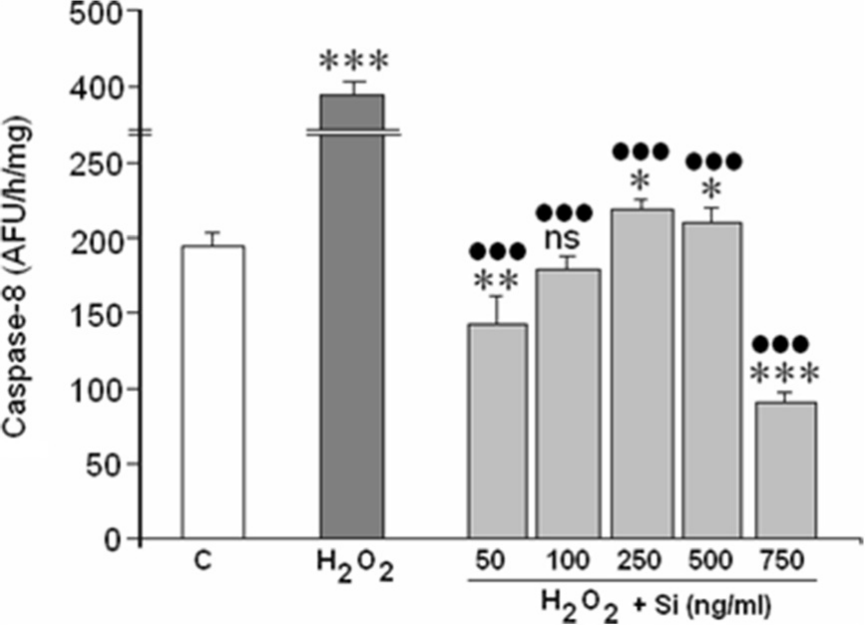
**Action of Si on caspase-8 activation mediated by H**_**2**_ **O**_**2**_ **.** (*)/ns = Statistical significances referred to the basal values. ns = no significant, (*) = p < 0.05, (**) = p < 0.01 and (***) = p < 0.001. (●) = Statistical significances between H_2_O_2_ in absence and presence of silicon G5. (●●●) = p < 0.001.

Figure 6 shows that exposure to 400 μM of H_2_O_2_ during 24 h further induced caspase-8 activation in the SH-SY5Y culture. Si G5, at concentrations between 100 to 750 ng/mL, completely reversed this H_2_O_2_- mediated effect, and reduced caspase-8 to lower than basal levels.

### Effects on TNF-α

Since caspase-8 activation is mediated though death receptor activation, TNF-α levels were measured by ELISA in H_2_O_2_ treated cells. There was not measurable TNF-α release mediated by H_2_O_2_ (data not shown).

## Discussion

H_2_O_2_ is a classic ROS that is normally produced in cells, including neurons [29, 30] and thus, it has been considered an appropriate molecule to study the possible protector effect of Si under cell death conditions in the human neuroblastoma SH-SY5Y cell line.

Our data clearly demonstrate that H_2_O_2_ induced a great cell viability lost (by about 40%), in agreement with Chetsawang et al. [31] who found SH-SY5Y cells loss viability mediated by H_2_O_2_. Those authors suggest that Ras protein could be involved in this cell survival decrease.

However, Si levels ranging 200 to 1000 ng/mL increased cell survival, confirming our hypothesis that Si can alleviate H_2_O_2_ neurotoxicity. At higher Si concentration (2000 ng/mL) did not protect cellular viability and this could be because Si may be toxic at high concentrations. Toxicity from high Si concentrations has been suggested by several researchers [32, 33]. Neuronal cell death by apoptosis induced by oxidative stress has already been reported [34] but acute oxidative stress also often induces necrotic outcomes [35].

**Figure 7:**
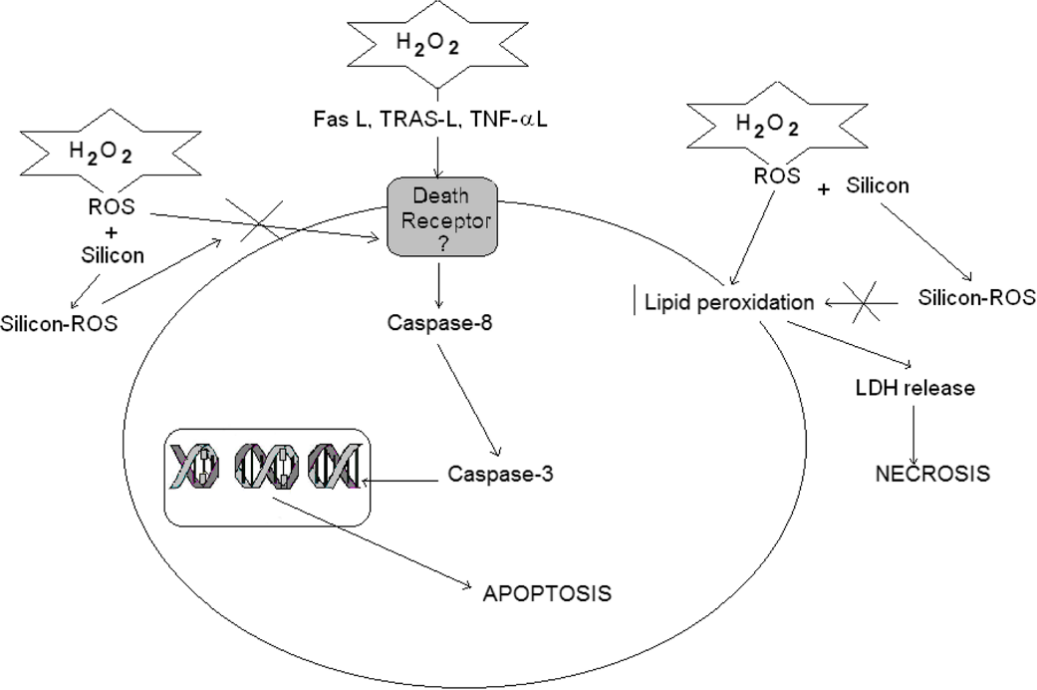
**Diagram of the mechanism by which both H**_**2**_ **O**_**2**_ **and Si induce cellular damage and neuroprotection.** H_2_O_2_ inducing apoptosis: H_2_O_2_, perhaps, by induction of death ligand, may activate death receptor responsible to activate caspase-8 (inductor enzyme) which activates caspase-3 (effectors enzyme). This last enzyme damages DNA and protein and as consequence produces apoptosis. H_2_O_2_ inducing necrosis: H_2_O_2_ (ROS) induces lipid peroxidation which conduces to LDH release and necrosis death. Si effect: In the presence of Si, H_2_O_2_ treated cells remove H_2_O_2_ (ROS) preventing neurotoxicity induced by it.

Oxidative stress, produced by excess ROS, is considered to be the major contributor to cell death in several CNS pathologies [36] such as Parkinson’s disease [37] and Alzheimer’s disease [38] or cerebral ischemia reperfusion after stroke [39, 40]. Since ROS may induce apoptosis or necrosis depending on the level of intracellular ROS and ATP levels [41], the mechanism by which H_2_O_2_ induces cellular death and the possible protector effects of Si against both types of cell death requires study. The results showed that the loss of cell viability induced by H_2_O_2_ in the SH-SY5Y was the result of both necrosis and apoptosis (Figure 7). With regard to the necrosis, H_2_O_2_ increased LDL release 4.5 times. Si, doses of 50 to 500 ng/mL, reversed the H_2_O_2_ induced LDH release on a great extent. However, the highest concentration of Si (750 ng/mL) employed did not exert any significant effect on cell necrosis death although it did reduce the caspase-3 activation. The protective effect of Si against H_2_O_2_ toxicity might be a consequence of its capacity to remove ROS because Si at doses ranging from 50 to 500 ng/mL completely prevented the ROS increase in H_2_O_2_ treated cells.

Moreover, regarding apoptotic death, H_2_O_2_ produced caspase-3 activation in the treated SH-SY5Y cells. This activation was not mediated via the mitochondria pathway but through the death receptors because caspase-8 but not caspase-9 was activated by H_2_O_2_. Effects of H_2_O_2_ as inductor of apoptosis or necrosis have been described in different cell types [42–45]. Generally, ROS-mediated apoptosis is linked to the intrinsic pathway through Bax activation, which aggregates into mitochondria and leads to cytochrome-c release. Consequently, this event activates caspase-9 which finally leads to caspase-3 activation. However, in our case we found no caspase-9 activation. On the other hand, apoptosis mechanisms acting through the extrinsic pathway have also been described in HeLa cells treated with different H_2_O_2_ concentrations for 8 h. Since apoptosis, via the extrinsic pathway, is mediated through death receptors, the possible involvement of TNF-α in cell death was investigated in our study. The results suggested that H_2_O_2_ was not able to increase TNF-α release under our experimental conditions. Nonetheless, this result does not mean that activation of caspase-8 cannot be mediated by death receptors other than TNF-α, such as Fas, TRAS, or DR4,5, which would probably also be involved. In this way, Medan et al. [46] reported that H_2_O_2_ and hydroxyl radical (OH^•^) were key mediators of apoptosis in macrophages, increasing FasL; and Wang et al. [47], using an elegant approximation to study the ROS species responsible for the implication of FasL in apoptosis induction, demonstrated that H_2_O_2_ and O_2_^•-^ were the ROS species that were most involved in this process.

As a whole, our findings suggest that H_2_O_2_ induction of cellular death may be explained according to the scheme in Figure 7. H_2_O_2_, by an unknown mechanism, perhaps induction of a death ligand, that would activate the death receptor responsible to activate caspase -8 (inductor enzyme) which activates caspase -3 (effector enzyme). This last enzyme would damages DNA and protein and as a consequence, produces apoptosis. On the other hand, H_2_O_2_ induces lipid peroxidation, which conduces to LDH release and necrosis death. In the presence of Si, treated cells seem to remove H_2_O_2_ (ROS), preventing the neurotoxicity induced by H_2_O_2_. Taken together these neuroprotective effects from Si could suggest a preventive role for Si in the context of neurodegenerative disease, including Alzheimer’s disease.

## Conclusions

- Oxidative stress promoted by H_2_O_2_ induces cellular death through two mechanisms: apoptosis and necrosis.
- Apoptosis mediated by H_2_O_2_ acts through the extrinsic pathways since caspase-8 is involved without affecting caspase -9 activation in H_2_0_2_ treated human neuroblastoma cells.
- Si G5 protects, to a great extent, against both cellular death mechanism in the human SH-SY5Y in the presence of H_2_O_2._
- ROS removal seems a principal mechanism of Si to protect SH-SY5Y cell line against oxidative stress mediated by H_2_O_2._

As a whole, these mechanisms suggest that Si G5 could be a neuroprotective agent that would help prevent cell death under vulnerability conditions associated to strong ROS production.

## Abbreviations

AFC: 7-amino-4-trifluoromethyl-coumarin
Al: Aluminium
AMC: Fluorochrome 7-amino-4-methyl coumarin
BHT: Butylated hydroxytoluene
H2DCF-DA: 2,7-dichlorodihydrofluorescein diacetate
DMEM: Dulbecco’s modified Eagle’s medium
DMSO: Dimethyl sulfoxide
DTT: Dithiothreitol
FBS: Foetal bovine serum
H2O2: Hydrogen peroxide
LDH: Lactate dehydrogenase
MDA: Malon-dialdehyde
MTT: 3-(4,5-dimethyl thiazol-2-yl)-2,5-diphenyltetrazoliumbromide
Si: Silicon
SiO2: Silicon dioxide
ROS: Reactive oxygen species
TBARS: Thiobarbituric acid reactive substances.
